# Supplementary material for: Complexome profiling of the Chlamydomonas psb28 mutant reveals TEF5 as an early PSII assembly factor
Source: Plant Cell. 2025 Mar 17;37(6):koaf055. doi: 10.1093/plcell/koaf055 (PMC12164590; doi:10.1093/plcell/koaf055)
Supplement: koaf055_Supplementary_Data [file koaf055_supplementary_data.zip › psb28_interactive.zip!/plots/intensity/qualityPlots/peptides/SULTR3_Cre09.g401293.t1.1.html]

### Cre09.g401293.t1.1

Trivial name: SULTR3  
SeqCoverage [median]: 13.40  
Mass: 115.29 kDa  
  
 

| Sequence | MissedCleavage | Mass [Da] | wtIntensity | muIntensity | Score |
| --- | --- | --- | --- | --- | --- |
| AAAAGAAATAAAAASVAAAPTAAEVAHAR | 0 | 2431.26 | 725.01 | 883.30 | 67.33 |
| AAAIVAAK | 0 | 713.44 | 1352.16 | 2271.62 | 77.75 |
| AVAAISQVGK | 0 | 942.55 | 0.00 | 285.88 | 52.72 |
| AVAATHTADPVQVLVLNLSR | 0 | 2074.15 | 1138.68 | 0.00 | 84.58 |
| GVQASAAGGR | 0 | 872.45 | 1958.69 | 2766.77 | 93.65 |
| GVQSALDR | 0 | 844.44 | 543.40 | 0.00 | 68.38 |
| LLDVLK | 0 | 699.45 | 419.08 | 0.00 | 94.67 |
| LPEGVPVPTEFLEK | 0 | 1553.83 | 412.44 | 0.00 | 55.35 |
| VIHAPFNELR | 0 | 1194.65 | 2333.43 | 14852.93 | 89.05 |
| VPVVDVSGLEVLEESAAELER | 0 | 2239.16 | 375.10 | 0.00 | 48.31 |
| VQSVSPSDASILLASSGNIK | 0 | 1972.05 | 455.55 | 0.00 | 75.67 |
|  |  |  | 9713.5 | 21060.5 |  |
